# Analysis of public perceptions on the use of artificial intelligence in genomic medicine

**DOI:** 10.1186/s40246-024-00686-6

**Published:** 2024-11-18

**Authors:** Jack E. Harrison, Fiona Lynch, Zornitza Stark, Danya F. Vears

**Affiliations:** 1 Department of Paediatrics, The University of Melbourne, Parkville, 3052 Australia; 2 Murdoch Children’s Research Institute, Parkville, 3052 Australia; 3 Melbourne Law School, The University of Melbourne, Carlton, 3052 Australia; 4 Victorian Clinical Genetics Services, Parkville, 3052 Australia; 5 Australian Genomics, Parkville, 3052 Australia; 6 Department of Public Health and Primary Care, Centre for Biomedical Ethics and Law, 3000 Louvain, Belgium

**Keywords:** Bioethics, Artificial intelligence, Qualitative, Machine learning, Genomics

## Abstract

**Purpose:**

Next generation sequencing has led to the creation of large pools of genomic data with analysis rather than data generation now the limiting factor. Artificial intelligence (AI) may be required to optimize the benefits of these data, but little is known about how the public feels about the use of AI in genomics.

**Methods:**

We conducted focus groups with members of the Australian public. Participants were recruited via social media advertisements. We explored potential uses of AI in genomic medicine, the benefits, risks, and the possible social implications of its use.

**Results:**

Participants (*n* = 34) largely felt comfortable with AI analysing their own genomic data and generally agreed about its benefits. Concerns were raised over data security, the potential for misdiagnosis, and bias AI may perpetuate. Many participants wanted checking mechanisms for when results were generated using AI.

**Conclusions:**

The insights gained from these discussions help to understand public concerns around the use of AI in genomic medicine. Our findings can help to inform both policies around genomic AI and how to educate the public on its use.

**Graphical abstract:**

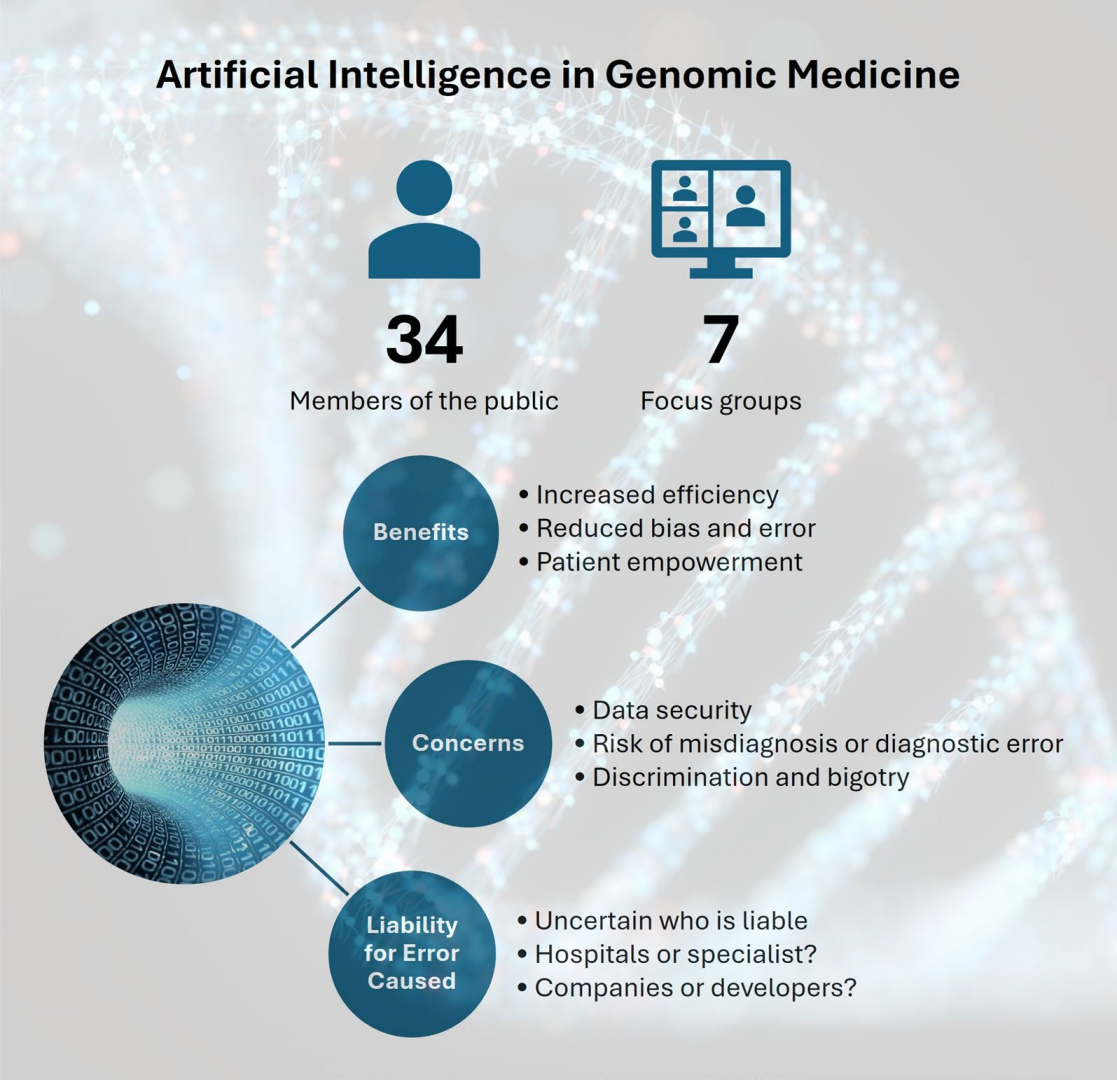

## Introduction

Large volumes of genomic data are now being generated as part of healthcare, with the ability to analyse the data in a timely manner becoming a limiting factor [1]. At the same time, our knowledge of the roles of genes and variants in health and disease continues to evolve at a rapid pace. Studies show, for instance, that although fewer than 50% of rare disease patients receive a diagnosis from initial genomic testing, reanalysis of existing genomic data following a period of two years can improve diagnostic yield by 10% [2]. A recent survey of Australian laboratories showed that of the 17,000 non-diagnostic genomic tests performed in Australia between 2018 and 2021, only 900 have been reanalysed (manuscript under review). At the current rate, it would take 85 years to complete a single cycle of reanalysis of these data; manual reanalysis is clearly not feasible.

Artificial Intelligence (AI) tools will therefore be necessary for the efficient analysis and reanalysis of genomic data. We define AI as computer systems able to perform tasks that would normally require human intelligence [3]. Many modern AI tools—such as ChatGPT and Autocorrect—are built through the process of machine learning (ML). ML utilises algorithms to iteratively learn from training data specific to the AI’s intended use, allowing the AI to find complex patterns in the data without explicit human direction [4]. Although not all AI are developed using ML [3], AI complex enough for the accurate diagnosis of disease is likely to require ML. While most of the public is familiar with the concept of AI, they are less familiar with the idea of AI in medicine [5]. Additionally, the public often link AI to concepts ranging from chatbots and video games to science fiction and popular media, which suggests there is potential for the public to hold misconceptions about how AI is used in genomic medicine.

Various AI tools have already been developed to identify potential pathogenic variants in genomic data [6, 7, 12]. Although meta-analysis of 29 studies found using AI in variant interpretation had no statistically significant impact on diagnostic yield over manual analysis alone [2], one particular tool was shown to improve diagnostic yield by greater than 15%, alongside reducing the analysis burden on human professionals by over 90% [6]. This shows great potential for use of AI in reanalysis of genomic data at scale.

Despite this, using AI in genomic analysis presents a range of ethical and practical challenges. For example, AI built from ML can lead to a ‘black box’ system where the reasons why an AI makes decisions are not explicable [8]. This lack of transparency makes it difficult to determine who should be held liable in the event of AI-related error [9]. Additionally, bias may be introduced into the AI, either through the datasets used for training or the people developing the algorithms [10]; in genomics, this could lead to higher rates of misdiagnosis for members of marginalised groups [11, 12]. While there are methods for debiasing AI, further research into their efficacy is still required. [10, 13]

Although research has explored public opinions about the use of AI in healthcare, little research has examined these perspectives specifically in relation to genomic medicine. Public trust is critical and hence it is important to understand public and professional perspectives on ethical issues to meaningfully engage in debate and inform the use of AI.

To address this gap, we aimed to explore how the public perceives the use of AI in genomic medicine, with a specific focus on its use in reanalysis of genomic data from undiagnosed patients.

## Materials and methods

### Sampling and recruitment

The study participants consisted of members of the public over the age of 18 years, living in Australia, and who spoke English competently. The study was advertised on Facebook via the Murdoch Children’s Research Institute Facebook page. The advertisement linked to a form where participants could enter their contact details. Screening calls were conducted by JH to gauge English competency and collect demographic data. Eligible participants were then invited to complete an online consent form and provide their availabilities in order to schedule the focus groups, which were then organised via email. Participants received instructions for the focus group and were asked to watch a 5 min video [14] which provided background information about the topics to be discussed. Participants received an AUD$75 (USD$50) gift card as reimbursement for their time after the focus group was completed.

### Data collection and analysis

The focus groups were semi-structured and facilitated by DV with assistance from JH who monitored the discussion, resolved technical issues, and polled participants on their opinions at the beginning and the end of each focus group.

We conducted and recorded focus groups lasting 1.5 h, in March 2023 using Zoom. The focus groups explored public perceptions on the use of AI in genomics with reference to the example of automated reanalysis. Participants were asked two polling questions at the start of the focus group about their level of comfort with AI analysing their own medical data (using MRI brain scan and genomic data as examples). The question about genomic data was repeated at the end of the discussion. The term “genomic data” was introduced to the participants during the pre-focus group video and was described as the result of genomic sequencing. The focus group guide can be found in Supplementary materials.

Interview transcripts were analysed using Inductive Content Analysis, which involves developing the codes from the texts themselves and comparing categories between other texts within the dataset, as opposed to coding based on a predetermined list [15]. Coding was carried out iteratively until all relevant data was coded into categories and subcategories. All transcripts were coded by JH; FL and DV co-coded a subset to ensure rigor.

## Results

### Demographics

Thirty-four members of the public participated across seven focus groups each with 4–6 participants. Participant ages ranged from 22 to 74 years (mean 38.6 years). Demographics can be found in Table 1.

**Table 1:** Participant demographic data (*n* = 34)

	Frequency	Percent (%)	Mean
Gender			
Man	8	23.5	
Woman	26	76.5	
Age			
18–29 years	9	26.5	38.6
30–39 years	14	41.2	
40–49 years	5	14.7	
50 years and older	6	17.6	
Area of residence			
Metropolitan	23	67.6	
Regional	6	17.6	
Rural	2	5.9	
Number of Children			
None	13	38.2	1.5
1	5	14.7	
2	7	17.6	
3	5	14.7	
4 or more	4	11.8	
Place of birth			
Australia	24	70.6	
Elsewhere	10	29.4	
Language Other than English Spoken at Home			
No	26	76.5	
Yes	8	23.5	

**Fig. 1:**
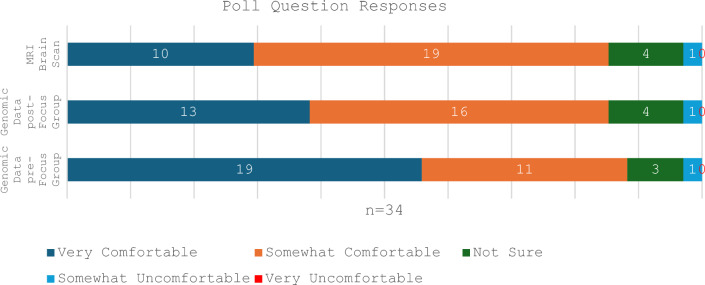
Distribution of participant answers to the question "How comfortable are you with AI analysing your own 'X' data," from 'Very Comfortable' to 'Very Uncomfortable’

Regarding the poll questions (Fig. 1), prior to the discussion, 29/34 participants (85.3%) indicated they were either ‘very comfortable’ or ‘somewhat comfortable’ with AI analysing both MRI brain scan and genomic data, although more were ‘very comfortable’ with AI analysing genomic data (13/34; 38%) than MRI brain scan (10/34; 29%). After discussion, 30 (88%) participants indicated they were comfortable with AI analysing genomic data, of which 19 (56%) answered ‘very comfortable’. One participant answered ‘somewhat uncomfortable’ for all three questions.

Qualitative analysis identified three categories: (1) Benefits of Artificial Intelligence in Genomic Medicine; (2) Concerns over Artificial Intelligence in Genomic Medicine; and (3) Liability for Error Caused by Artificial Intelligence.

Representative quotes are used to illustrate these categories. An ellipsis (…) represents where a large portion of text has been removed, and square brackets ([]) represent where a word has been replaced for clarity or to protect the anonymity of participants. Quotes are labelled in parentheses based on the order that they occur (e.g., (Q1) refers to quote one). Participants are given pseudonyms based on their focus group number (e.g., F1P1 refers to focus group one, participant one).

### Benefits of artificial intelligence in genomic medicine

Participants discussed many potential benefits of using AI in genomic medicine (Table 2). Participants mostly agreed on the possible benefits of AI in genomic medicine, stating that the ability to process a greater volume of data much faster than manual analysis would result in faster turnaround times for patients, more diagnoses, and reduced workload for professionals (Q1).

**Table 2:** Illustrative quotes for benefits of AI in genomic medicine

AI will be more efficient than manual analysis	Q1:*“Yeah, I think, speed is one thing, especially for time and sensitive diseases where you really cannot afford to wait many, many years. You know, go through a lot of sessions *etc*. So, I think speed is one thing. So, to me, these are things that will help, like to be sharing data making [the AI] more accurate and then being able to take on a lot of the workload which a human being couldn’t catch up with.”* [F2P1]
AI will reduce bias and error	Q2:*“Just in general I think probably hopefully making more diagnoses and interpreting it more volume of data in a shorter amount of time and hopefully without that bias that we have from humans, as you said like if you’re specifically looking for something or like making an accurate diagnosis. I think that that’s probably the best way that AI’s probably going to help us just. More diagnoses over a shorter amount of time and hopefully with a greater accuracy.”* [F1P2]
AI will empower patients	Q3: *“… I think you get a lot of that from doctors going “nah, you don’t have that and stuff like that”, “it’s fine”. But realistically it’s not and I’ve been there. I know that’s how I found out I have a TIA because I took myself to the hospital and they sent me home saying “no you’re fine” which really isn’t good enough. And then I went to a neuro, and he sent me for an MRI and we got exactly what had happened … So, I definitely think the AI would remove that part anyway and hopefully you’re not waiting around kicking stones for testing, you’re actually getting it then.”* [F1P1]
Trust in genomic AI	Q4: *“…coming from a diagnostic community and a personal background and the experience of just overwhelming problems with bias in clinical professionals and cognitive dissonance and all kind of other human problems with coming to the correct conclusions. I actually trust the computers as a starting point more than I trust the people….”* [F7P2]

Even though misdiagnosis and accuracy were commonly cited concerns about the use of AI, many participants perceived the potential for AI to reduce bias and human error to be one of its greatest benefits (Q2).

Participants expressed trust in genomic AI, attributing this trust to several factors, such as AI’s potential to help them on their own diagnostic odyssey or empower other patients on theirs (Q3). Others recounted their own negative experiences with medical professionals; one participant discussed how their experience with human error and misdiagnosis led them to be more trusting of AI analysis than manual analysis (Q4).

### Concerns over artificial intelligence in genomic medicine

Participants noted several concerns with genomic AI (Table 3). Some participants expressed distrust for the analysis of genomic data with AI. Sometimes this was expressed more as a gut feeling of distrust towards computer analysis than it was relating to any concrete concern (Q5). Other participants indicated that their distrust was due to the technology still being in the early stages of development and that they may be more trusting of AI analysis in the future (Q6). Participants generally agreed that the results of AI analysis should go through a checking mechanism or periodic auditing, usually by a human professional, before it was handed back to patients (Q7).

**Table 3:** Illustrative quotes for concerns over AI in genomic medicine

Distrust in genomic AI	Q5:*“…even though that’s just an emotional thing because it defies logic, if the AI’s better at the job than a human why do I still want a human to check it? And that’s just, I think, an emotional tie to human intelligence that I have. [It] doesn’t necessarily make sense, it’s not logical, it’s just how I feel.”* [F3P3]
	Q6:*“Me, I was not sure about either of them. Mostly because at this stage the technology is still very much in its infancy. We’re still, [AI] is still being trained and so, we’re not 100% sure what’s going on.”* [F5P3]
	Q7:*“I sort of feel the trust with computer devices isn’t there yet and I want some sort of failsafe in place where I knew that maybe once in a while it was being audited in some way. Where it was checked against a human to make sure that the program was doing what it was meant to be doing and if it was equally as accurate as a human then I wouldn’t have an issue with it at all.”* [F1P2]
Data security	Q8:*“The data breach because of the hacker nowadays. If it starts to become like Medibank, they were attacked by a hacker and they stole the client’s data, the customers data, I think that’s a big concern of how safe our data is going to be kept by these—whatever company runs the AI.”* [F1P4]
	Q9:*“Well, I guess the thing is, with insurance you don’t want to say, “Oh, I want to insure myself with whatever company and they go, “Oh well due to” and they might not even say it. They might just go, “oh sorry we can’t cover you for this.” And you know, fob you off and then you find out later that they had this information, and they thought no that’s too big a risk because of whatever this information is giving them and so that could potentially you know, open a can of worms there …”* [F7P1]
	Q10:*“You get DNA testing to find out information so it might be a bit of risk to you, where in order to search for that information. So, I mean yeah, it’s not ideal, it’s not great. No one wants to be hacked. But if what you get out of completing the test is so meaningful, I think it’s a risk you might be happy to wear. It wouldn’t deter me from doing the test.”* [F5P1]
Risk of misdiagnosis or diagnostic error	Q11:*“AI is new, maybe there’s not a lot of data backing it up so like, how correct is the analysis going to be? You know, what if they get it wrong like if you get your results and they’re wrong how does that change the course of your life potentially. You could be getting extremely impactful information and then if it’s wrong that could have a huge impact on someone.”* [F6P2]
	Q12: *“…The second concern that I had and forgive me because my understanding of AI is quite rudimentary. Is, are humans training the AI? Because if they’re training the AI then are they going to build in the biases that already exist in the health care system. Where, you know, for example, a lot of health studies, particularly in the past were just based on male participants and not suitable for females…”* [F3P3]
Discrimination and bigotry	Q13:*“… as a bisexual person, I’m really uncomfortable with the idea of, if we discover a gay gene or anything because I know with a lot of other things that are perfectly ok, the minute that there’s a genetic explanation for it, it’s immediately eugenics with a lot of people, and it’s immediately like, “Oh, well now we can get rid of it. We can get rid of the autism. We can get rid of all these other things that don’t need to be gotten rid of.” And I’m kind of petrified of that kind of thing being discovered and then being turn against LGBTQ people. Against, you know, whoever would be the victim of that kind of attitude.”* [F3P4]
	Q14: *“I’m also worried if it leaks into the dating world like say for example somehow a dating app got a hold of this information and you’re indirectly being discriminated against like there might be people on that app that want to have kids but they look at people’s genetic information and then they’re like, “well I’m not even going to try that person.”…”* [F6P2]

Discussions about data security were prevalent across the focus groups, with several participants citing recent high profile data breaches in Australia (Q8). These discussions often centred on how a genomic data breach might affect their insurance policies or employment (Q9). In contrast, several participants stated that they did not care what happened to their data as long as it was able to help them or their children (Q10).

Another common concern was the risk of misdiagnosis or error from AI analysis and how that might impact patients (Q11). Many participants expressed concern about how the bias of the researchers developing the AI, or the datasets used to train the AI, might lead to bias in the AI itself, and subsequently misdiagnoses for patients (Q12).

Participants discussed how certain groups—such as people from the LGBQTI + community or people with disabilities—may be discriminated against. One participant stated how, as a bisexual person, they were concerned about how discovery of the genetic causes of bisexuality might lead to discrimination against them (Q13). Additionally, some participants expressed concern about being rejected romantically by prospective partners for their genetic make-up (Q14).

### Liability for error caused by artificial intelligence

Many participants were interested to know who would be liable in the event of a misdiagnosis or other error related to the use of AI (Table 4). Some participants expressed that how AI works with ML to make its own decisions independent of its original coding may mean no one can be held accountable for these errors (Q15), while others wanted to know who they should sue for damages (Q16).

**Table 4:** Illustrative quotes for liability for error caused by AI

Not sure who is liable	Q15:*“I guess the problem with that is once the AI’s set up, even if it’s set up in the most perfect manner with[ML]; it can change. So, it’s not really the issue with the creator of the algorithm itself but it’s what it’s been fed and how it’s developed. So, I really don’t know who would be to blame for [a] diagnosis that is incorrect. Which is why I guess you would need a person on the end to kind of check for results and put some checks and balances before giving the diagnosis to the patient themselves.”* [F7P3]
	Q16: *“…let’s say the AI says “Oh, you have this, you’ve got this” and the doctors agree to it right but then the outcome turns out to something else, and you get upset and you want to sue somebody. Who do you sue? Do you sue the specialist? Do you sue the AI makers? Do you sue the algorithm? …”* [F2P1]
Hospitals or specialist are liable	Q17: “… *But then if someone using that scalpel uses it wrong, cut somebody incorrectly and they die as a result then it’s that person’s responsibility so you know along those lines I would think if the machine and it’s outputs or its program are not fundamentally flawed but you as a clinician in a forward way apply its output and the error was really in your application of it then it was really your responsibility. So, if it spits out a diagnosis that’s abjectly incorrect for that patient but the reason it got that wrong is you didn’t give it the right information then it’s not its fault it’s your fault.”* [F7P2]
	Q18: *“I was going to say, like, if I got incorrect information and I know it happens like doctors misdiagnose all the time. I would assume that the person that’s responsible, I’m assuming this information comes through New South Wales Health, so it would be like the doctor or the hospital, or maybe there’s like some procedure put in place that if you get a diagnosis that somehow it’s checked like it’s confirmed because I think if you get a misdiagnosis it’s New South Wales Health or the doctor that is responsible for that or the pathologist that somehow stuffed up the test or result.”* [F6P2]
Companies or developers are liable	Q19: *“I think it’s circumstantially dependent. It depends, what you mean by blame, like whether you mean legal liability or responsibility or more just like who should fix it. But I think if there’s a fundamental structural programming in the program that can and will repeatedly spit out wrong things then the creator or custodian or however it works, you know, of the program themselves has to have some responsibility. The way you would if you were producing a scalpel or an X-ray machine or you know any other equipment you’ve got to make sure it does its job reliably …”* [F7P2]

Some participants referred to how AI is just a tool meant to aid in diagnosis and indicated that it is the professionals or institutions using these tools that are liable. This suggests that how the genomic AI is used should determine liability (Q17). Participants also related this idea to their desire for a human checking mechanism (Q18).

Some participants stated they thought the company or institute developing the AI should be held accountable for any errors. This often tied into their concerns about how bias could be embedded in AI by developers or datasets. Following this logic, participants felt that in some circumstances (such as an embedded error) the developer would be liable, whereas in others (such as a hospital or specialist using AI as a tool to aid diagnosis) those hospitals or specialists could be deemed liable (Q19).

## Discussion

To our knowledge this is the first study taking an in-depth look at the views and preferences of the public on the use of AI in analysing genomic data for medical purposes. Building an understanding of these perspectives is crucial for the development of automated tools and to ensure that the use of these tools aligns with the interests of the public.

While there is previous research exploring public views on the use of AI in a healthcare setting [5, 16, 17], any research exploring perspectives on these issues has been in the context of medicine broadly, with no discussion of the use AI in the reanalysis of genomic data. As such, how these previous studies relate to the use of AI in genomics is difficult to determine. Previous research indicates that genomics—as with other ‘omics’ fields—suffers from a lack of public understanding [18], making it even more critical to explore public perspectives.

### Comfort with artificial intelligence in genomic medicine

In line with other studies [5, 16, 17, 19], our findings suggest that the Australian public has a generally positive view on the use and development of medical AI tools. Our participants perceived the greatest benefits of AI in genomic medicine to be reductions in the time and cost required for analysis; increased volumes of data available for analysis; possible improvements in accuracy of analysis; and empowerment of patients, as found by others [5, 17]. However, participants were still concerned about the accuracy of AI and how their data might be stolen or misused. Similarly, our participants expressed a strong desire for human oversight of AI either by periodic auditing or review by a specialist, which is also in line with findings of previous research. [5, 17]

These findings suggest that the public’s view of AI in genomics is not substantially different from their view on the use of AI in other areas of medicine [5, 17]. However, the difference in our participants’ views between MRI brain scans and genomic data suggest that for some, the type of data being analysed can affect the level of trust in AI use. This aligns with the findings of Middleton et al. [20], who found in their research on collection and storage of genomic data that some participants described genomic data as being ‘special’ or ‘different’ compared to other forms of medical data. Further research is needed to clarify how the type of medical data may affect patient attitudes to AI analysis.

### Impact of artificial intelligence on accuracy and bias in genomic medicine

Interestingly, the potential impact of AI on the accuracy of genomic results was viewed by participants as both a benefit and a concern. On one hand, AI may be able to see things in the data that humans cannot, increasing the accuracy of analysis and subsequently the diagnostic yield. On the other hand, depending on how it is programmed and trained, AI might be less likely to produce accurate results than humans. This is in line with previous research on public perspectives of AI in healthcare more broadly [5, 17], where participants were also found to hold these seemingly contradictory opinions.

Similar to our findings with members of the public, the potential for AI to reduce bias and improve accuracy of diagnoses in healthcare is often cited as a benefit by physicians and patients [15, 20, 21]. However, there is an increasing body of research demonstrating how AI can inflate and perpetuate bias in medicine against already marginalised communities [11, 12]. In terms of bias, participants in our study believed that AI systems could be programmed to *not* incorporate the bias that human professionals have. However, they also recognised how bias could be unintentionally embedded into AI algorithms by their developers.

Participants discussed how the datasets used to train the AI—not just the algorithm the AI is built on—also had the potential to introduce bias into these systems. Yet researchers have proposed that the incorporation of bias into data collection, preparation, and AI development can be mitigated by having datasets that are as large and diverse as possible, being transparent about the demographic characteristics of the datasets, taking de-biasing steps in development of automated systems, and continual learning of AI systems as data is updated [10]. Our findings suggest that the public agrees with these researchers on how bias should be mitigated in AI development.

### Impact of artificial intelligence on the security of genomic data

AI development and use in any medical context requires the collection and sharing of a large amount of medical data. This can lead to multiple issues surrounding data security and data autonomy for patients. Accordingly, how genomic data is stored was a prominent concern amongst participants in our study; they discussed their desire to know how their data is being used and distrust of companies that may be profiting from their data, referencing several recent high-profile data breaches. These findings echo concerns from previous research on public perspectives of medical AI [17] and storage of genomic data [20, 23, 24]. However, it contrasts with another study which found data security to be a relatively minor concern when compared with the infancy of the technology and distrust in AI companies [19]. In line with previous research [25], several of our participants described how they felt less concerned with how their data was stored or used because of their previous medical experiences; instead, they were more interested in how genomic testing and AI may help them or their families.

Despite their concerns for data security, participants recognised the need for genomic data to be accessed and shared broadly for the improvement of AI diagnostic tools and for genomic medicine as a whole. Participants described how large and diverse datasets are needed to build accurate systems for diverse populations. This suggests that members of the public may be less concerned about the sharing of genomic data if they know it is going to help others.

### Liability for error caused by artificial intelligence in genomic medicine

ML can lead to the development of a ‘black box’ system, which means that it may not always be clear how or why an AI model gave the results that it did [4, 8]. It is also possible that through this process, AI systems may produce results that are outside of our current understanding of the relationship between genetics and health outcomes. In these cases, health professionals may not be able to determine whether the results from AI are accurate or should be trusted. If these results were given to a patient and later found to be inaccurate, it would be difficult to determine who would be liable for the possible harm that is caused. Participants in our study were interested to know who would be held accountable for these kinds of errors. However, when posed with this scenario, many participants were unsure, noting that if AI makes its own decisions, responsibility would be difficult to assign.

Discussions amongst our participants around the issue consider that liability may be dependent upon how the error occurred and the purpose of using the technology. Participants related genomic AI to other medical devices; if another medical device was to cause an error due to how it was designed, then it would be those responsible for building the device that would be liable for the error. Conversely, if someone were to use the device in a way that were unintended, or misinterpret the results that were given, then that individual would be liable. No clear preference for who should be held liable was found in our discussion, which contrasts with the findings of Khullar et al., [26] who found a strong preference by the public for physicians to be held liable for errors caused by medical AI over the developers of the tool. This area requires further research to determine more precisely how the public believes liability for error caused by genomic AI should be determined.

The aim of this research was to examine perspectives from a diverse sample of the Australian public, rather than a representative one. As such, some perspectives (e.g., parents of children with genetic conditions) may be overrepresented in our data, whereas others (e.g., men) are underrepresented. The findings of our research could be used to help build a survey on the use of AI in genomic medicine, which could more easily receive a larger and potentially more representative pool of respondents. Future surveys of this nature should be mindful to ensure that participants have sufficient understanding about what AI is and the contexts in which it could be used in genomic medicine, without biasing their perspectives. While focus groups did discuss broad implications of AI in genomic medicine, the material used to inform participants on the topic prior to discussion focused primarily on AI in the context of reanalysis of genomic data. While this made the concepts discussed more tangible for participants, and aided understanding, our findings may not be as applicable to other uses of AI in genomic medicine, such as image-based evaluation of faces or MRI scans to determine gene-phenotype relationships. Similar future studies with a more direct focus on these specific uses of AI in genomic medicine should be performed to gain a more accurate depiction of the public’s perspective.

As the use of genomic sequencing and periodic reanalysis of genomic data become routinised, the need for AI tools to analyse this growing volume of data is quickly becoming apparent. Given this, it is important to understand how the public perceives the use of AI in genomic medicine to ensure that AI implementation aligns with the public’s interests and that any concerns, both warranted and unwarranted, are addressed before we expect it to be broadly accepted. Importantly, error and bias in AI systems need to be minimised through the inclusion of large diverse datasets and de-biasing systems. In addition, AI use should be constantly monitored and checked against professionals regularly to ensure accurate results. Further research is needed to determine how the public values these aspects of AI in genomic medicine relative to each other when trade-offs are required.

## Ethical approval

This study was reviewed and approved by The Royal Children’s Hospital Human Research Ethics Committee (HREC ID: 90814). Participants provided voluntary, informed consent.

## Data Availability

The datasets generated and analyzed during the current study are not publicly available to protect the privacy of the participants but are available from the corresponding author on reasonable request.
